# Supplementary material for: The Connections among Interacting with Nature, Nature Relatedness and Dietary Choices: A Pilot Mixed Methods Study
Source: Int J Environ Res Public Health. 2024 Jul 10;21(7):899. doi: 10.3390/ijerph21070899 (PMC11276622; doi:10.3390/ijerph21070899)
Supplement: Supplementary file 1 [file ijerph-21-00899-s001.zip › Supplemental Table S1.pdf]

Table S1: Themes, categories, and exemplar quotes

Theme	Categories	Exemplar Quotes
The influential role of Nature Relatedness	Interacting with nature	“I enjoy walking a lot and so I spend a good portion of my days like just walking and observing my surrounding environment and I think that that's like that's been like my favorite way to enjoy and explore nature.” – P07<br><br>“My son engages more like playing with sticks, digging up stuff and finding rocks and more actively engaged in hands on nature exploration, picking up bugs. So a lot of that is secondary through him. The bug he picks up or whatever… He’s very intrigued about the natural world so that gets me more hands on involved than I would otherwise be.” – P23<br><br>“So I love to birdwatch. Just seeing the animals, you know.” – P05<br><br>“I would say that I have plenty of plants inside my living space. Yeah, they're they're just right next to me. [laughter] Yeah yeah, but they are they're close to me and I would say like prior to the pandemic, I don't think that this was something like as important to me like this was something -- this was only a recent change as I was spending more time indoors that I found was like important to cultivate. But prior to that, I would say that I had no nature indoors.” – P07

	Creating or reflecting on memories and experiencing wonder	"It's weird to describe it but the smell – not the smell of nature, but the fresh air. Sometimes I get nostalgic, and it reminds me of my childhood where I would – some memory of me being outside in the fresh air, it'll remind me of that. I smell cut grass and I think of my dad cutting grass in the summer while I was playing baseball or something with my neighbors. Things like that." – P10
		"I could sit in the chair all day. No bathroom, just read my book, people watch, just watch the water. Just nature itself is utterly amazing. Just how we are able to survive here and the water and the creatures and the tide, there's just so much you can sit there and just take in. How do we survive here? Its amazing." – P11
	Sustainable dietary choices	"I think environmental like consciousness, is something that is important to me. Like finding foods that are sustainable, maybe like sustainably sourced, and have like a lower like environmental impact on the community that I live in is pretty important. So like I think I tend to eat more vegetarian focused meals as a result." – P07
		"We don't eat a lot of meat and if we do eat meat, its like chicken or ground turkey. For health reasons and climate reasons. We try and eat mostly fruits and vegetables if possible." – P24

Finding harmony in oneself	Physical Health	*"What initiates these walks?*<br><br>Like health, just like wanting going like a healthy walk for like your body and everything. Getting some fresh air, definitely." – P29<br><br>"Most commonly is running. I choose a running path that is a more natural path – as much natural engaging as possible. So its mostly running along the [river] and through some parks along the way. So I do that pretty commonly." – P23<br><br>"I think just to help have an extended life and fewer. I don't know fewer illnesses. I mean, I'm healthy and don't take any medications. And don't have any issues with anything and I would like to live as long as I can without having to be really at the doctor a lot so.<br><br>And I know that I feel better when I'm eating that way just because. You know, the more natural the foods are, the more healthy you feel the less my joints ache or I mean, I can tell when I eat a lot of like, if I have a kind of greasy pizza. I can just tell that my whole my palate feels swollen and it just is salty. And anyway, so just trying to stay away from foods that make me feel yucky." – P31<br><br>"Overall, I know foods aren't supposed to be labeled as bad and good. But I try avoid having a super high diet of saturated fats, trans fats, a lot of cholesterol, things like that. I try and stay away from more processed foods and rely more on whole foods that I cook. Sometimes its hard. It's more convenient to eat

		those types of things but overall, I try and eat everything in moderation that is high and bad foods." – P10
	Mental Health	"[Nature is] my saving grace. When I go through breakups or bad times or that's where I, you know -- I tried meditation, which I can't really get there 'cause I'm like all over the place sometimes. And yoga, I do do that. But uhm, nature, that's where it's at for me. Yeah. Brings me down." – P05
Connecting to others through nature	Being in nature facilitates current relationships	"If the weather is good, I might participate in playing Frisbee with my friends, apartment friends, or badminton with apartment friends. Walking I already stated. Or maybe if we are planning something maybe like kind of a potluck or maybe BBQ etcetera. So, if the weather is good, maybe participate in those activities so it's more like eating and enjoying. And everyone loves eating, especially when it comes to snacks rather than your regular food. … Maybe sometimes kayaking." – P26<br><br>"Generally when I'm walking … my neighbor and I are doing it together." – P09<br><br>"Helping my parents with their gardens just now. I know what to make them happy when I spend time with them out there and like they like gardening. So it's something that will make them happy if I help them up." – P29

	Being in nature facilitates past relationships	“I have a lot of like fond travel memories so like going to Niagra falls and like my grandma seeing the falls for the first time and like I think that was really special for her. And like just having all of these visits that we've done to different places, I've traveled a lot with my family, so I think it's also holds significance of like being with my family and like doing those things together.” – P29<br><br>“In my kitchen, I'm looking right now, I have some tiles of sunflower seeds, I have a photograph that my late uncle took of a yellow chrysanthemum. And he had it framed, and he gave it to my grandmother, which was his mother. And when she passed away, I decided that I would take it and give it a new home.” – P09
